# Whether hysteroscopy improves fertility outcomes in infertile women: a meta-analysis and systematic review

**DOI:** 10.3389/fendo.2024.1489783

**Published:** 2024-12-16

**Authors:** Yidi Wang, Zunhao Tang, Chanchan Wang, Xiuxiang Teng, Junqin He

**Affiliations:** ^1^ Beijing Obstetrics and Gynecology Hospital, Capital Medical University, Beijing Maternal and Child Health Care Hospital, Beijing, China; ^2^ College of Traditional Chinese Medicine, Shandong University of Traditional Chinese Medicine, Jinan, China; ^3^ Department of gynecology, Tongxiang Traditional Chinese Medicine Clinic in Chaoyang District, Beijing, China; ^4^ Department of gynecology, Beijing Hospital of Traditional Chinese Medicine Affiliated to Capital Medical University, Beijing, China

**Keywords:** infertility, hysteroscopy, IVF/ICSI, meta-analysis, pregnancy

## Abstract

**Purpose:**

Infertility is affecting more and more couples of appropriate age. Hysteroscopy (HSC) has certain effects on the uncompleted pregnancy and live birth caused by uterine microenvironment. Based on the evidence, this paper systematically evaluates the effectiveness and safety of HSC intervention on the fertility outcome of female infertility.

**Methods:**

Randomised controlled trials (RCTS) of hysteroscopy intervention in female infertility were included in the literature database. The retrieval time was from the establishment of the database to December 10, 2022. RevMan 5.4 software was used for statistical analysis to study the effects of HSC on clinical pregnancy rate, live birth rate and abortion rate.

**Results:**

A total of 14 RCTS were included. Five studies evaluated the effect of HSC on live birth rate, and HSC had an overall positive effect on live birth rate. Fourteen studies evaluated the effect of HSC on clinical pregnancy rates, and preoperative HSC was associated with significant improvements in pregnancy rates for both first-time IVF/ICSI patients and repeat IVF/ICSI patients. Eight studies showed no significant difference in the effect of HSC on miscarriage rates.

**Conclusion:**

As a visual examination/treatment technique, HSC can improve the clinical pregnancy rate and live birth rate in most studies, while the risk of abortion is within the acceptable range, and can be used as a recommended examination method for infertile women.

## 1 Introduction

Infertility is a fertility disorder caused by a variety of causes. Infertility is the third major disease after tumor, cardiovascular and cerebrovascular diseases, and the incidence in developing countries is higher than that in developed countries (1). The findings of the latest report from WHO show that 1 in 6 people globally are affected by infertility in their lifetime (2).

Failure to establish a clinical pregnancy after 12 months of regular unprotected sexual intercourse, or due to impaired fertility in the individual or with their partner, is called infertility (3). Infertility can be divided into primary and secondary infertility according to whether the woman or the man has a clinical pregnancy history with his spouse. According to the etiology, it can be divided into female factor infertility, male factor infertility and unexplained infertility (4). The causes of female infertility mainly include ovulation disorders and pelvic factors. Poor ovulation function accounts for about 15% of all infertile couples and 40% of female infertility (5). Reduced ovarian reserve, anatomic, endocrine, genetic, functional, or immune abnormalities of the reproductive system, chronic diseases, and sexual conditions incompatible with coitus are also causes.

Assisted reproductive technology (ART) is one of the most important medical breakthroughs of the 20th century. For infertile couples, it undoubtedly brings more possibilities for them to be able to have their own children. In ART, the success rate of pregnancy is related to many factors such as patient age, embryo quality, endometrial receptivity and intrauterine environment, among which the intrauterine environment and embryo quality are particularly important. At present, most patients can have high-quality embryos for transfer, but the clinical pregnancy rate is still not satisfactory. Ultrasonography (US), especially transvaginal ultrasound (TVUS), can be used to screen women for possible ovarian, endometrial, or uterine abnormalities and to examine fertility problems. This assessment can be enhanced by hysterosalpingogram (HSG) and saline infusion/gel instillation sonography (6, 7). However, because the above method is indirect examination, it is easy to miss diagnosis and misdiagnosis for mild uterine abnormalities, and the nature of uterine lesions cannot be clearly defined. Relatively speaking, hysteroscopy technology has the advantages of intuitive, accurate, convenient pathological biopsy and surgical treatment. With the development of hysteroscopy technology over the decades, the complications of the procedure have become fewer and the safety has greatly improved. Due to the close connection between hysteroscopy technology and the development of technology (camera technology, micro uterine lens, photo imaging, expansion media, etc.), the current technological development has been enough to meet the needs of hysteroscopy. Hysteroscopy has gradually become the “gold standard” test for evaluating the uterus because it can directly show the uterine cavity and its associated pathological diseases and treat any abnormalities found.

Nevertheless, a practical question remains: for intrauterine assessment, does hysteroscopy, as the gold standard, improve reproductive outcomes relative to ultrasound or saline infusion (8)? When there is clinical evidence, hysteroscopy can be used as part of the initial examination of infertility patients, but it is not the first examination, because its effectiveness in improving reproductive outcomes has not been determined (9).

Therefore, scholars have been reassessing the clinical significance of hysteroscopy in the diagnosis and treatment of infertility in terms of uterine factors and its role in the examination of infertility.

Zhang HY compared the effect of hysteroscopic polypectomy treatment and no treatment on pregnancy outcomes of patients receiving ART (10).​ Mao XY explored whether hysteroscopy could improve IVF outcomes for patients with recurrent implantation failure (RIF) before the start of the IVF cycle (11). In addition to studying the effect of hysteroscopy on the pregnancy outcome of infertile patients undergoing assisted reproductive technology, some scholars have also studied the effect of hysteroscopy on improving the reproductive outcome of infertile couples. Yang studied the effect of diagnostic hysteroscopy on reproductive outcomes in infertile women without intrauterine lesions (12).

This study aimed to conduct a meta-analysis of the latest randomised controlled trials to evaluate the efficacy of hysteroscopy in improving reproductive outcomes in infertile couples. Because hysteroscopy may help improve reproductive outcomes.

Given the potential for diagnostic and/or surgical hysteroscopy to improve reproductive outcomes at different stages of the diagnostic and therapeutic efforts of infertile couples, we included all available randomised controlled trials (RCTs), whether diagnostic hysteroscopy or concurrent surgical hysteroscopy, or a second surgical hysteroscopy in infertile women diagnosed with abnormal uterine cavity. Similarly, we included patients who underwent hysteroscopy prior to their first attempt at standard IVF or ICSI, and those who underwent hysteroscopy prior to their next IVF/ICSI attempt after one or more failed IVF/ICSI attempts.

## 2 Methods

### 2.1 Inclusion criteria

(1) Study type: all studies had to be randomised controlled trials (RCTs);
(2) Subjects: All infertile women with or without uterine cavity abnormalities diagnosed by ultrasound (US), salpingography (HSG), or SIS/GIS, registered during basic infertility testing (including IUI), and before being a candidate for any ART, Infertile women in their first attempt at IVF/ICSI or who have experienced one or more failed IVF/ICSI attempts;
(3) Intervention: Experimental group intervention: diagnostic or surgical hysteroscopy was performed during the first infertility examination or before the first or subsequent ART attempt (IVF/ICSI). Control group: no hysteroscopy was performed before the first or second IVF/ICSI attempt.
(4) Outcome measures: Primary outcome measure: live birth rate (LBR), defined as delivery of a live baby after 20 weeks of gestation resulting in at least one live birth. Births resulting from singleton births, twin births, or multiple pregnancies were counted as a live birth.

Secondary outcome: clinical pregnancy rate, defined as the detection of one or more gestational sac by means of ultrasound visualisation or the diagnosis of pregnancy by confirmed clinical signs of pregnancy; Miscarriage rate, defined as spontaneous abortion of clinical pregnancy before 20 complete weeks of gestation; Procedure-related complications, defined as any complications arising from hysteroscopy.

### 2.2 Exclusion criteria

(1) Intervention measures: previous use of other treatment regimens or combined with other treatment regimens were excluded; (2) Duplicate publication, incomplete data, and inability to obtain the full text.

### 2.3 Data sources

PubMed, The Cochrane Library, Embase, China National Knowledge Infrastructure (CNKI), VIP, Wanfang and Chinese Biomedical Literature Database (SinoMed) were searched. The search time was from the establishment of the database to December 10, 2022. In addition, the references of the included articles were searched to supplement the acquisition of relevant information. The search took the form of a combination of free words and subject words. Take the Pubmed database as an example:

((“Pregnancy Rate”[Mesh]) OR (Rates, Pregnancy)) AND (((((((“Hysteroscopy”[Mesh]) OR (Hysteroscopies[Title/Abstract])) OR (Uterine Endoscopy[Title/Abstract])) OR (Uteroscopy[Title/Abstract])) OR (Uteroscopies[Title/Abstract])) AND ((“Infertility”[Mesh]) OR (((((“Sterility, Reproductive”[Title/Abstract]) OR (Sterility[Title/Abstract])) OR (Reproductive Sterility[Title/Abstract])) OR (Subfertility[Title/Abstract])) OR (Sub-Fertility[Title/Abstract])))) AND ((randomised controlled trial[pt] OR controlled clinical trial[pt] OR randomised[tiab] OR placebo[tiab] OR drug therapy[sh] OR randomly[tiab] OR trial[tiab] OR groups[tiab]) NOT (animals[mh] NOT humans[mh]))).

With the above search terms as keywords, according to the characteristics of different databases, comprehensive search is carried out in subject, title, full text, etc.

### 2.4 Data extraction

Two investigators independently extracted data according to the inclusion and exclusion criteria, and a third party assessor participated in the discussion and decision in case of disagreement. The extracted information included general characteristics of the literature (first author, region, year, literature type, etc.), treatment regimens, diagnosis and treatment standards, and outcome indicators.

### 2.5 Quality evaluation

The literature was evaluated according to the “risk of bias assessment tool” used in Cochrane systematic reviews. The evaluation included randomised sequence generation, allocation concealment, blinding, completeness of outcome data, selective outcome reporting, and other biases. The results were expressed according to high risk of bias, unclear risk of bias, and low risk of bias.

### 2.6 Statistical methods

Revman5.4 statistical software provided by Cochrane Collaboration was used. relative risk (RR) and 95% confidence interval (CI) were used as statistics for binary variables. The weighted mean difference (WMD) or standard mean difference (SMD) and its 95% confidence interval (CI) were used as statistics for continuous variables. The *Q* test was used to analyse the statistical heterogeneity of the included studies, and I^2^ statistic was used to evaluate the statistical heterogeneity among the included studies. When there was no heterogeneity or the heterogeneity was small (I^2^ ≤ 50%), the fixed effect model was used for Meta-analysis. If there was a large heterogeneity (I^2^ > 50%) between studies and the clinical heterogeneity was not obvious, the random-effects model was used for meta-analysis. When there is significant heterogeneity, the source of heterogeneity should be analysed.

### 2.7 Grade of evidence

We also assessed the overall quality of evidence for the primary outcome using the GRADE approach, which takes into account not only issues related to internal validity but also external validity, such as directness of results (i.e., agreement between the populations, interventions, or outcomes measured in the studies actually found and those considered in our systematic review), inconsistent results between the included studies, and the lack of consistency in the findings. Imprecise results due to small sample size or few included studies, publication or outcome reporting bias.

## 3 Results

### 3.1 Results of literature screening

**Figure 1:**
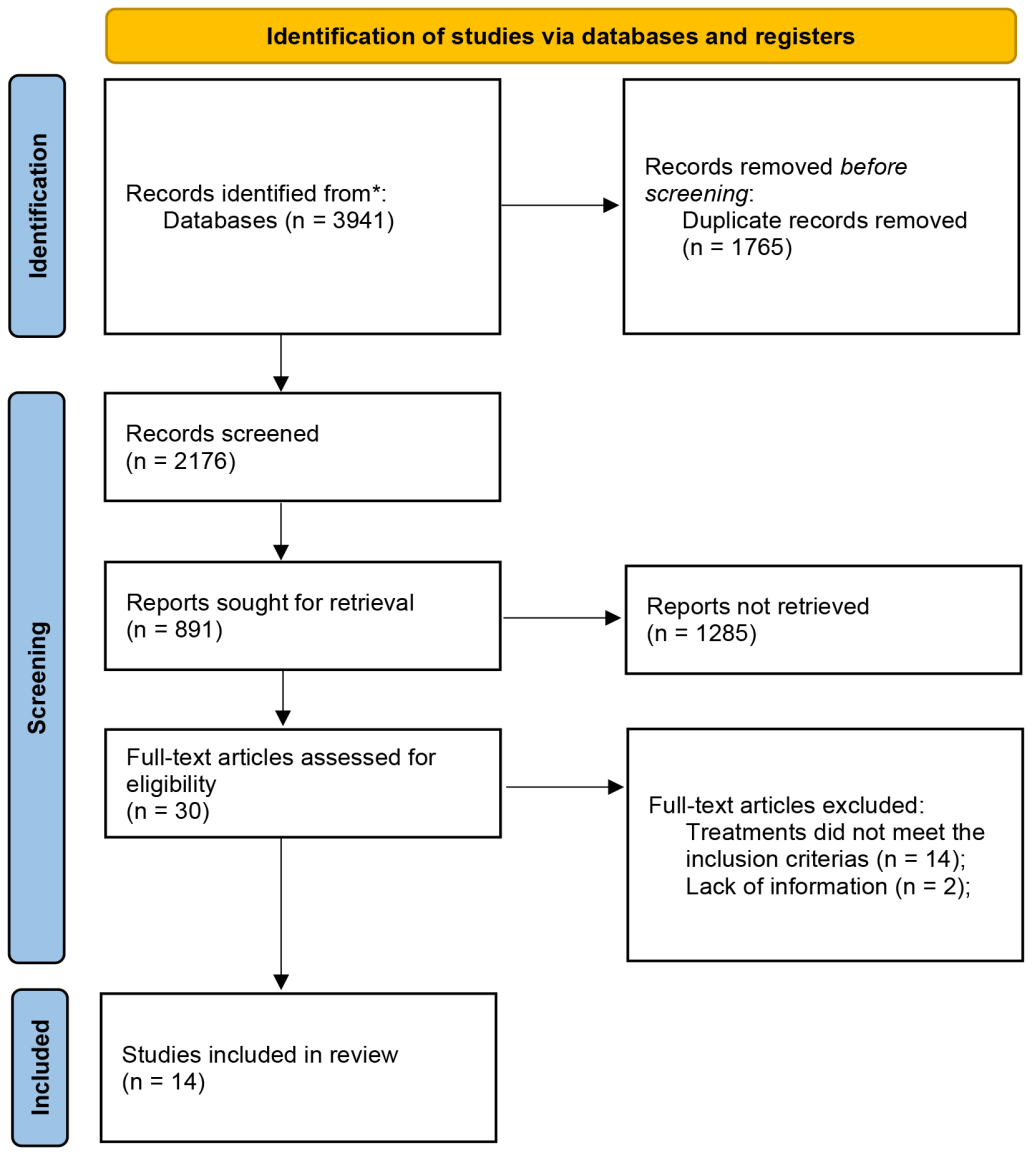
Flow diagram of literature retrieval..

Literature search results: A total of 3941 articles in Chinese and English were screened out. The PubMed, The Cochrane Library, Embase, CNKI, SinoMed, Wanfang and VIP were 109, 30, 102, 871, 909, 1490 and 430, respectively. After removing duplicates, 2176 articles were left. Finally, 14 literatures met the inclusion criteria, including 10 English literatures and 4 Chinese literatures, as shown in **Figure 1** (13–26).

### 3.2 Basic characteristics of the included studies

#### 3.2.1 Comparison of patient types and interventions

Six studies included infertile women undergoing IVF-ET/ICSI for the first time. (13, 14, 16, 18, 19, 25); two studies included women after their first failed IVF-ET (20, 26); and three studies included primary infertile women with two or more failed IVF-ET cycles (15, 17, 21); three studies included infertile women without mentioning whether they had undergone assisted reproductive technology or not. (22–24).

#### 3.2.2 Timing of hysteroscopy

In Hu (18) and Li (19), hysteroscopy was performed and treated accordingly before the first IVF-ET cycle, followed by an IVF-ET cycle. In Elsetohy (16) and Smit (25), hysteroscopy was scheduled in the early-mid follicular phase (days 3-12) of the menstrual cycle, and ICSI was performed within 3 months of hysteroscopy. In Alleyassin (14), hysteroscopy was performed on days 18 to 22 of the menstrual cycle. In Abid (13), diagnostic hysteroscopy was scheduled at mid-follicular stage. In Smit (25), hysteroscopy 1-3 months before starting IVF treatment. In Mei (20), patients underwent hysteroscopic electrosurgery at 3-7 days after the end of the menstrual period 1 month before freeze-thawed embryo transfer. In Wu (26), patients were examined using hysteroscopy 2-7 d after the patient’s menstrual period was cleared. In Demirol (15), all office hysteroscopies were performed 2 to 6 months after the last failed IVF cycle by the same physician. In El-Toukhy (17), outpatient hysteroscopy was performed within 14 days of menstruation and the IVF treatment cycle was started within the following month according to the standard IVF protocol. In Shawki (23) and Rama Raju (21), ICSI was performed after office hysteroscopy. in Shokeir (24) a single, site-specific endometrial injury was performed under hysteroscopic guidance from day 4 to day 7 of the menstrual cycle.

#### 3.2.3 Countries

Four studies were conducted in China, four studies were conducted in Egypt and one each in Tunisia, India, Netherlands, Turkey and Iran. One was a multicentre study conducted in seven centres in the UK, Italy, Belgium and the Czech Republic. For a detailed description of the included studies, see **Tables 1**, **2**.

**Table 1:** Basic characteristics of the included studies.

Studies	Country	Cases(T/C)	Inclusion Criteria	Treatment group interventions	Control group	Outcomes
Abid2021 (13)	Tunisia	84/87	Infertile women were eligible for this trial if they were scheduled to their first IVF. All patients were younger than 40 years, having regular cycles (28–32 days per cycle), having a normal uterine cavity as attested by normal systematic TVUS transvaginal ultrasound and HSG (absence of intra-uterine pathologies such as polyps, fibroids or septa), having FSH level less than 10 UI/l and an antral follicular count ≥12. All patients had a BMI ranged from 19 to 30 Kg/m^2^ and had given their oral consent after being clearly informed.	Patients were scheduled for diagnostic hysteroscopy in the mid-follicular phase. IVF was immediately started the next cycle if hysteroscopy was normal.	Immediate IVF.	Primary outcome: clinical pregnancy rate (CPR) after first fresh embryo transfer and resulting in a live birth rate (LBR). Secondary outcomes: implantation rate after first fresh embryo transfer, miscarriage rate, multiple pregnancy rate, duration of hysteroscopy and side effects (Visual analog scale and discomfort).
Alleyassin2017 (14)	Iran	110/110	Women who had underwent their first ICSI cycles.	The intervention group underwent office hysteroscopy before ICSI cycles. All women in the intervention group underwent office hysteroscopy between the 18th and 22nd day of their menstrual cycles.	The control group did not undergo office hysteroscopy before ICSI cycles.	The primary outcome was clinical pregnancy rate.
Demirol2004 (15)	Turkey	210/211	Four hundred and twenty-one patients who had undergone two or more failed IVF cycles, in which two or more good quality embryos transferred, participated prospectively in the study.	Patients had office hysteroscopic evaluation of the uterine cavity and cervix before commencing controlled ovarian stimulation for IVF treatment.	Patients did not have office hysteroscopic evaluation before commencing controlled ovarian stimulation for IVF treatment.	Mean number of mature oocytes; Fertilisation rate; Number of clinical pregnancies; Number of first trimester abortions.
Elsetohy2015 (16)	Egypt	97/96	Subjects with primary or secondary infertility candidate for ICSI by various indications were scheduled for a first IVF/ICSI treatment cycle with no abnormality detected, apart from intramural myomas without uterine cavity deformity, during transvaginal ultrasonographic examination performed during the follicular phase of the menstrual cycle were included.	ICSI with hysteroscopy. Hysteroscopic examination was scheduled in the early–mid follicular phase of a menstrual cycle (day 3–12).	ICSI without hysteroscopy.	Pregnancy rate.
El-Toukhy2016 (17)	The UK, Belgium, Italy and the Czech Republic.	350/352	Women were eligible if they were younger than 38 years of age; had a normal transvaginal ultrasound assessment of the uterine cavity; reported previously having two, three, or four fresh or frozen IVF treatment cycles ending in an embryo transfer but no pregnancy; and were undergoing a further treatment cycle of IVF (with or without intracytoplasmic sperm injection). Women aged 37 years were eligible to participate only if they had at least eight oocytes retrieved in the previous IVF cycle.	Women had an outpatient hysteroscopy within 14 days of menstruation and started the IVF treatment cycle in the following month according to a standard IVF protocol.	Receiving no hysteroscopy before starting their IVF treatment cycle.	The primary outcome was livebirth rate. Pre-specified secondary outcomes were rates of pregnancy, clinical pregnancy, embryo implantation, and miscarriage. We also recorded abnormal hysteroscopy findings and hysteroscopy-related adverse events. A health economic evaluation was planned and integrated into the trial design.
Hu2012 (18)	China	80/80	(1) Infertility factors are mainly female tubal factors; (2) primary infertility with >5 years of infertility or secondary infertility with >3 years of infertility; (3) no past history of IVF-ET assisted pregnancy.	Hysteroscopy is performed and treated accordingly before the first IVF-ET cycle, followed by an IVF-ET cycle.	First IVF-ET without hysteroscopy.	(1) Hysteroscopic findings and corresponding management. (2) Pregnancy outcome (graft implantation rate, clinical pregnancy rate)
Li2015 (19)	China	78/78	(1) Infertility factors are mainly female tubal factors; (2) primary infertility with >5 years of infertility or secondary infertility with >3 years of infertility; (3) no past history of IVF-ET assisted pregnancy.	Hysteroscopy and its treatment are given before in vitro fertilisation-embryo transfer is performed.	IVF-ET was performed directly without hysteroscopy related examination.	(1) Hysteroscopic findings and corresponding management. (2) Pregnancy outcome (graft implantation rate, clinical pregnancy rate)
Mei2021 (20)	China	50/50	(1) Patients with diverticulum secondary to cesarean section were diagnosed as infertile based on their medical history, physical examination and imaging MRI or (and) ultrasound; (2) Patients with diverticulum had a second frozen-thaw embryo transfer after their first failed IVF-ET; (3) Patients with diverticulum had at least one quality embryo transferred in a fresh cycle without pregnancy; (4) Endometrial thickness ≥8 mm on the day of transfer; (5) Good health status, clinical physical examination and laboratory There were no obvious abnormalities in clinical physical examination and laboratory examination. (6) FET transfer of 1 high quality embryo.	Luteal support protocol was given in both groups. Patients underwent hysteroscopic electrodesiccation at 3-7 days after the end of their menstrual period 1 month prior to the frozen-thawed embryo transfer.	Luteal support protocol was given in both groups. Routine freeze-thaw transplantation under abdominal ultrasound guidance.	(1) HCG positive rate, clinical pregnancy rate, early miscarriage rate, twin pregnancy rate, ectopic pregnancy and number of monozygotic twins. (2) Follow up for 6 months after treatment to observe the safety indexes of both groups.
Rama Raju2006 (21)	India	255/265	Patients who had undergone two or more failed IVF cycles, in which two or more good quality embryos were transferred per procedure, participated prospectively in this study.	Patients had office hysteroscopic evaluation prior to ovarian stimulation for IVF treatment.	Without office hysteroscopic evaluation prior to ovarian stimulation for IVF treatment.	Clinical pregnancy rate; Miscarriages rate; Live birth rate
Seyam2015 (22)	Egypt	100/100	Women previously diagnosed as unexplained infertility.	Receiving office microhysteroscopic procedure.	Without office microhysteroscopic intervention.	Pregnancy outcome.
Shawki 2012 (23)	Egypt	120/120	Asymptomatic infertility women (normal HSG and TVS)	Patients underwent ICSI after performing office hysteroscopy using non-touch vaginoscopic technique.	Patients were subjected to ICSI without office hysteroscopy.	Clinical pregnancies; Implantation rate
Shokeir2016 (24)	Egypt	60/60	Women with unexplained infertility for at least 1 year and defined as unable to conceive despite regular intercourse were enrolled.	Women were assigned to undergo a single, site-specific endometrial injury guided by hysteroscopy between days 4 and 7 of the menstrual cycle (CD4–CD7)	Receiving no intervention.	Primary outcome: cumulative clinical PR per woman. Secondary outcome: the first trimester miscarriage rate.
Smit2016 (25)	Netherlands	373/377	Women were eligible for the trial if they were infertile, scheduled to start IVF or ICSI treatment, and had a normal transvaginal ultrasound of the uterine cavity (defined as no visible intracavitary pathology—eg, submucous myomas, polyps, or septa)	Women were scheduled for hysteroscopy in the early to midfollicular phase of the menstrual cycle in an outpatient setting without anaesthesia, 1–3 months before the start of IVF treatment.	Immediate IVF.	Primary outcome: ongoing pregnancy within 18 months of randomisation and resulting in livebirth. Prespecified secondary outcomes: cumulative rates of implantation and miscarriage and the prevalence of intrauterine abnormalities. We also aimed to assess cost calculations and patient preference and tolerance of the procedures.
Wu2019 (26)	China	58/58	Patients with infertility who are to undergo IVF-ET treatment again.	In the observation group, hysteroscopy was applied on the basis of the control group. Hysteroscopy was applied to the patients 2-7 d after the patients' menstruation.	The control group was given conventional IVF-ET routine preoperative screening modality examination and treatment.	(1) To count the number of cases of endometrial polyps, uterine adhesions and anomalies in the two groups. (2) To compare the pregnancy success rate of patients in the two groups.

**Table 2:** Reported hysteroscopic findings in the intervention group.

Studies	Hysteroscopy group findings
Demirol2004 (15)	Normal:154 Abnormal:56 Endometrial polyps:33 Filmy and mild endometrial adhesions:18 Cervical adhesions:5
Elsetohy2015 (16)	Normal:55 Endometrial polyp:9 Submucous myoma:7 Cervical stenosis:6 Intrauterine adhesion:6 Uterine septum:6 Polypoid endometrium:4 Arcuate uterus:2 Unicornuate uterus:2
El-Toukhy2016 (17)	Cervical abnormalities: 14 Uterine cavity abnormality: 34 Subtle endometrial abnormality: 41
Rama Raju2006 (21)	Normal: 160 Polyps: 32 Stenosis: 30 Endometrial hyperplasia: 12 Synecheae: 12 Septate uterus: 8 Fibroids: 1
Seyam2015 (25)	Normal: 70 Endometrial polyps: 20 Submucous fibroids: 3 Intrauterine adhesions: 3 Polypoid endometrium: 3 Bicornuate uterus: 1
Shawki 2012 (23)	Normal: 70 Endometrial polyp: 11 Endometrial polyp: 4 Intrauterine adhesion: 4 Intrauterine adhesion: 7 Intrauterine adhesion: 1 Endometritis: 2 Endometrial hyperplasia: 3 Atrophic endometrium: 2 Others: 1

### 3.3 Risk of bias in included studies

**Figure 2:**
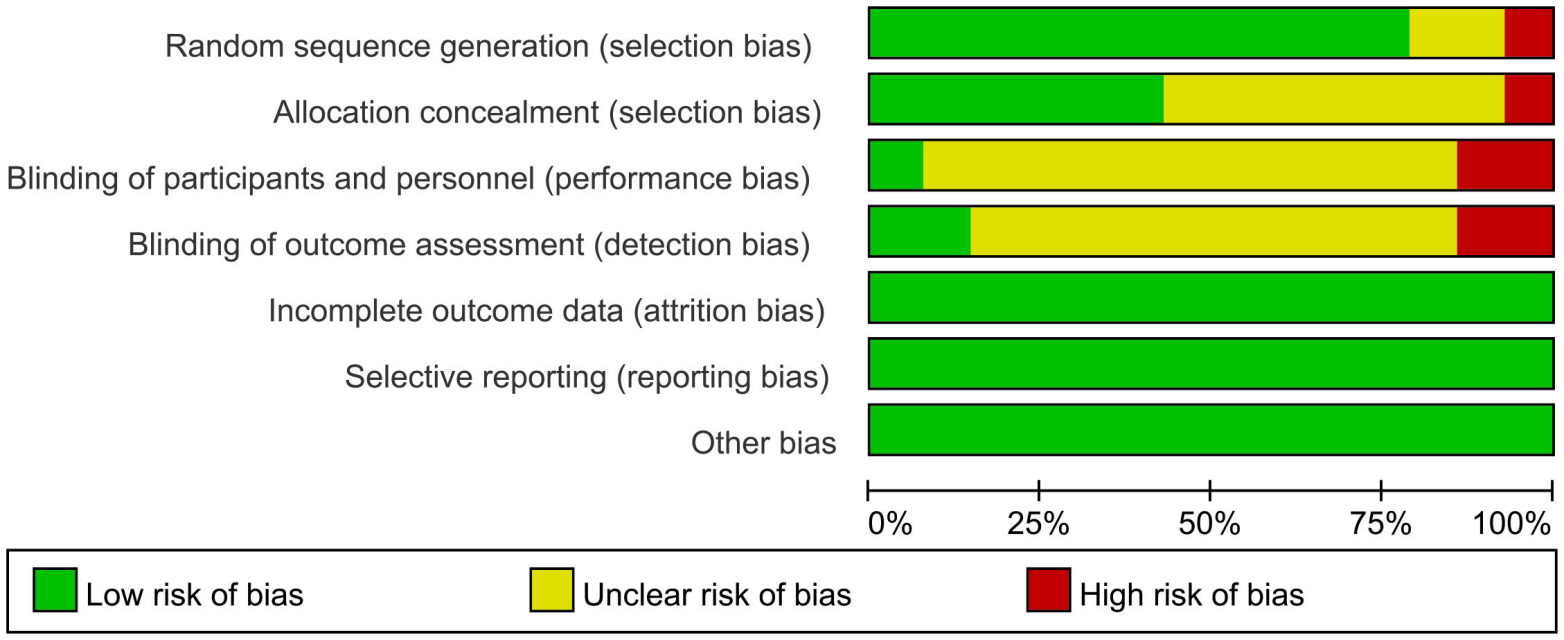
Risk of bias.

**Figure 3:**
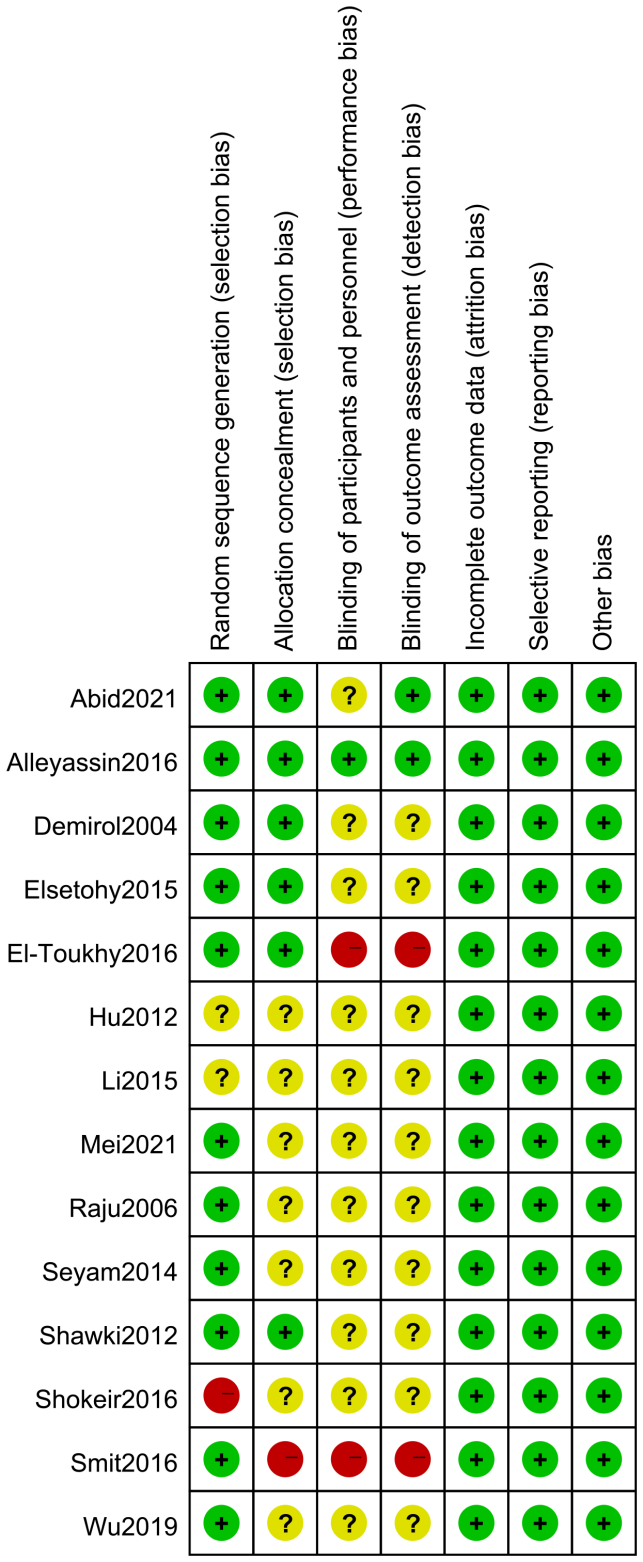
Risk of bias summary.

See **Figures 2**, **3**.

### 3.4 Outcome of the intervention

#### 3.4.1 Live birth rate

**Figure 4:**
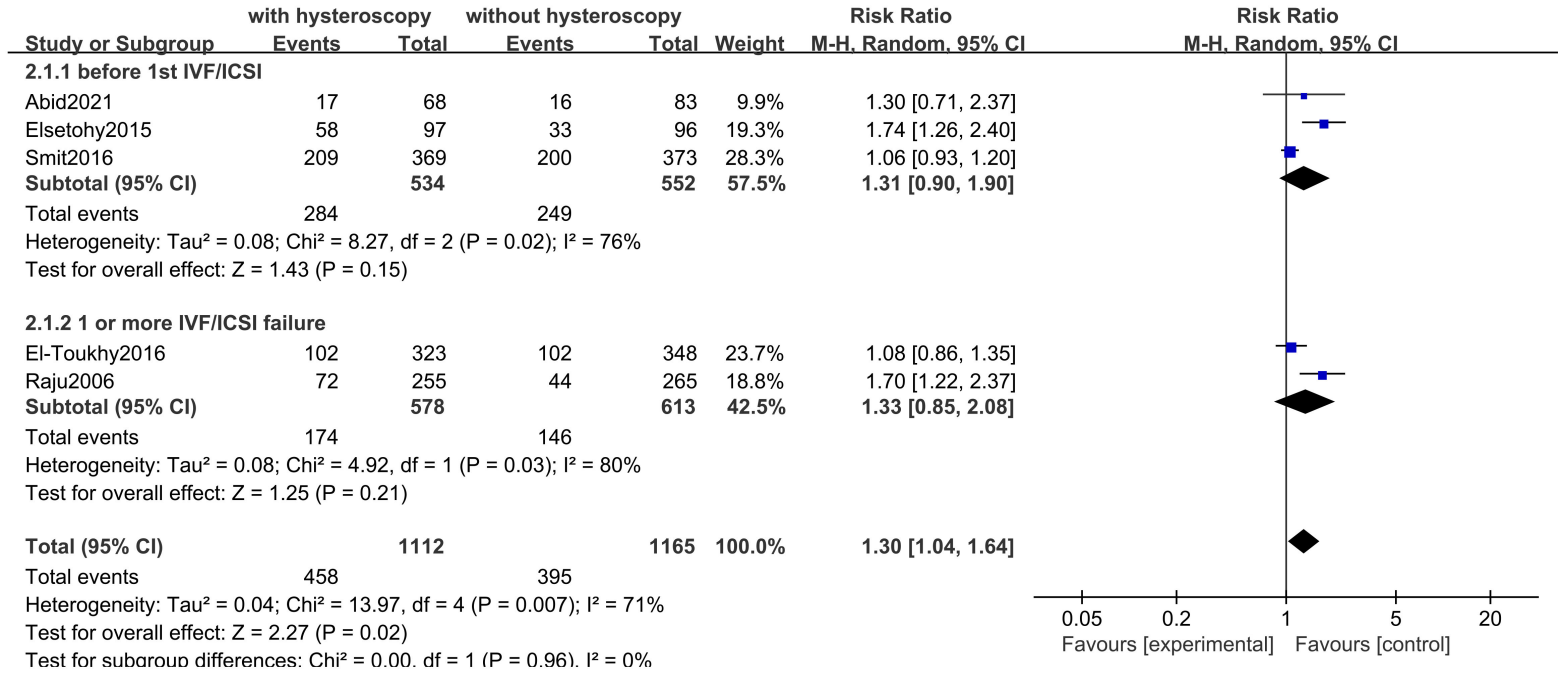
Forest plot of live birth rates.

**Figure 5:**
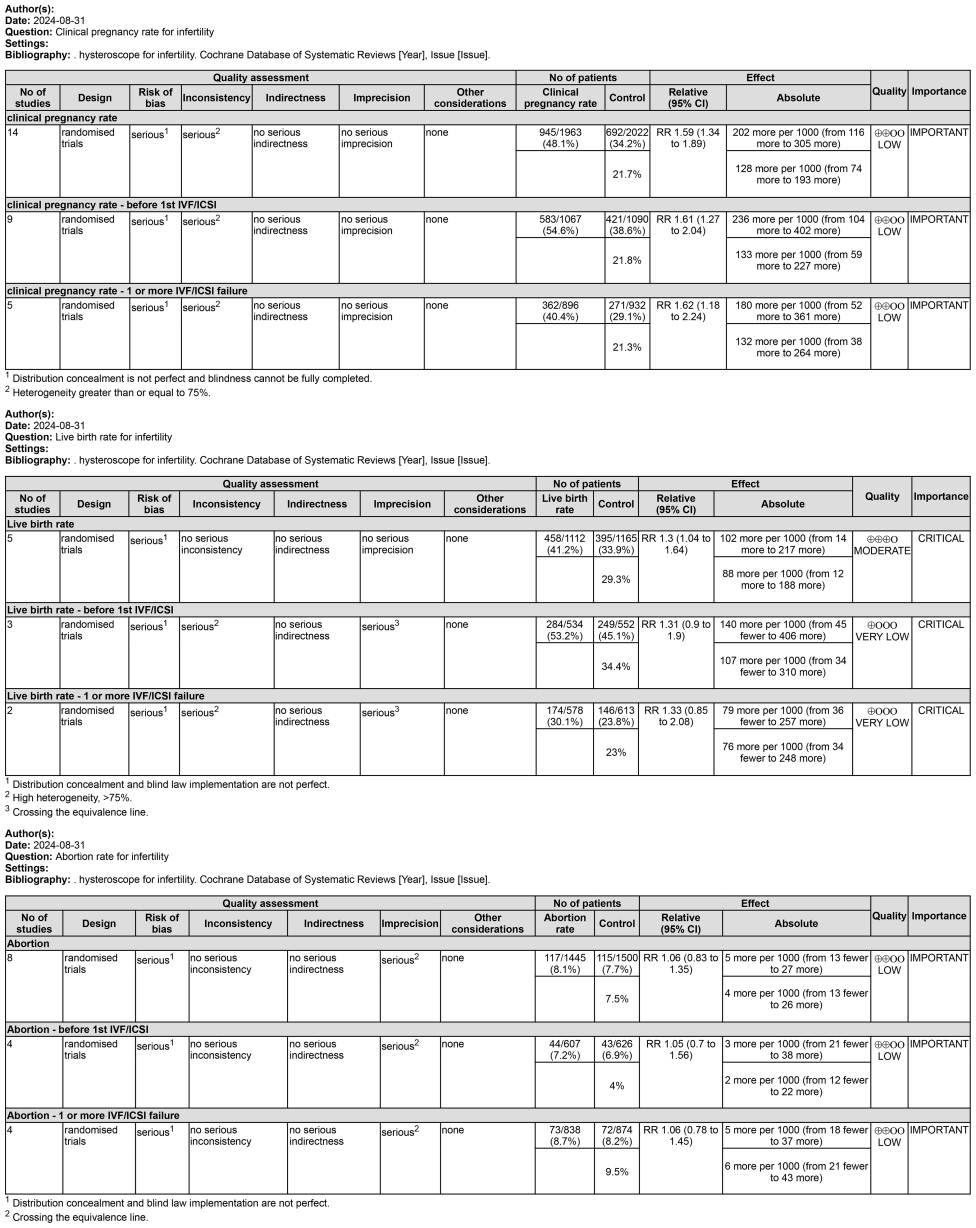
Results of evidence quality.

Only five studies assessed this result. Five studies of 2,277 subjects found that LBR was higher in the hysteroscopy group than in the control group (RR 1.30, 95% CI 1.04-1.64, I^2^ = 71%, *P*=0.007), and the difference was significant (*P*=0.02<0.05) (see **Figure 4**). The quality of the evidence was judged to be moderate. Three of the studies reported on the outcomes of women who underwent an attempt at hysteroscopy before their first IVF/ICSI, and in a subgroup analysis of 1077 women who underwent hysteroscopy before their first IVF/ICSI procedure, we found that hysteroscopy was superior to the non-hysteroscopy group (RR 1.31, 95% CI 0.90-1.90, I^2^ = 76%, *P*=0.02), but the difference was not significant (*P*=0.15 > 0.05). The quality of the evidence was judged to be very low (see **Figure 4**). In a subgroup analysis of 1191 women with implantation failure (one or more) after IVF/ICSI reported in 2 studies, hysteroscopy was similarly found to be superior to the non-hysteroscopic group (RR 1.33, 95% CI 0.85-2.08, I^2^ = 80%, *P*=0.03), but the difference was not significant (*P*=0.21>0.05). The quality of evidence was judged to be very low (see **Figure 5**).

#### 3.4.2 Clinical pregnancy rates

**Figure 6:**
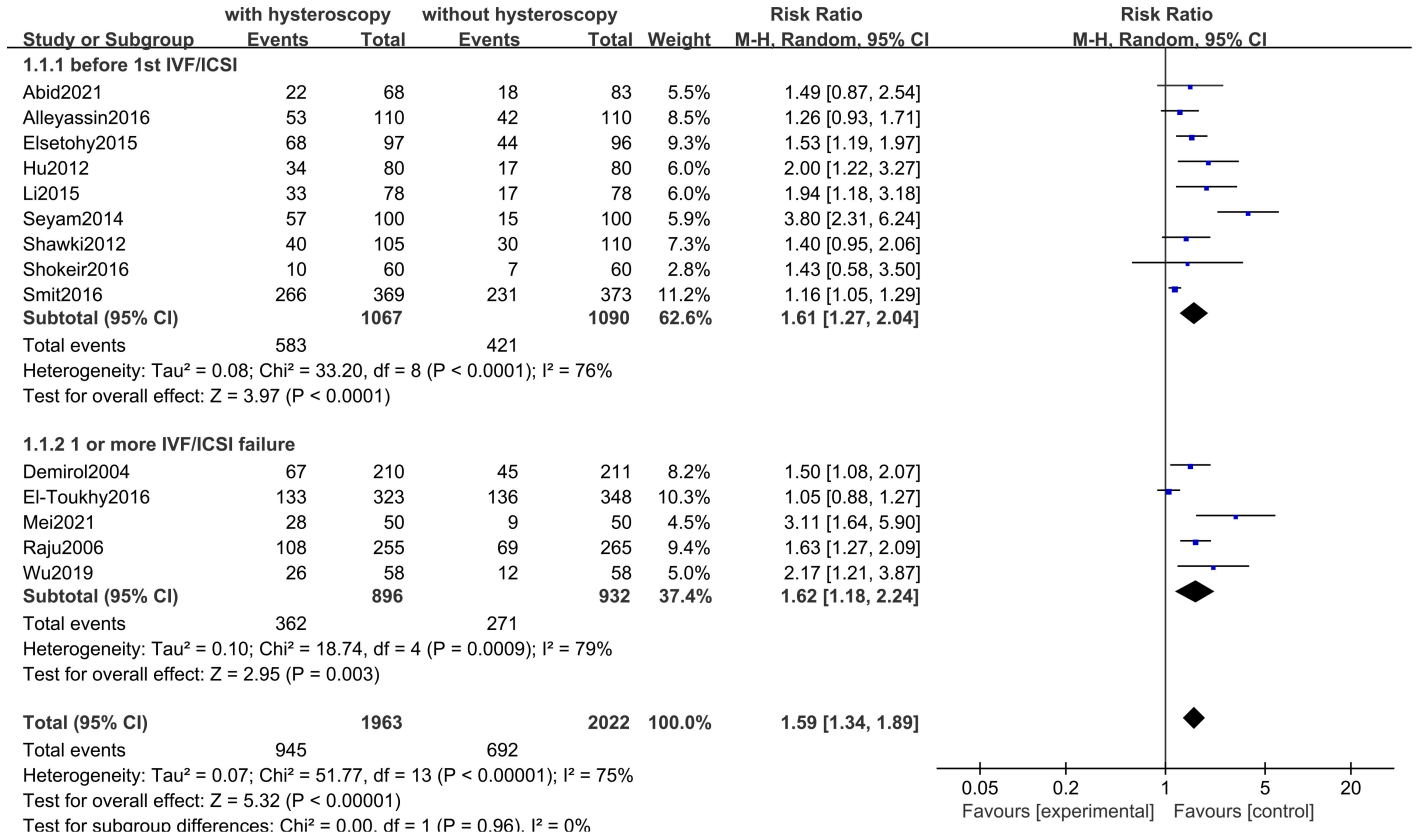
Forest plot of pregnancy birth rates.

Fourteen studies including 3985 participants. Hysteroscopy (RR 1.59,95% CI 1.34-1.89, I^2^ = 75%, *P*<0.00001) was superior to hysteroscopy with a significant difference (*P*<0.00001)(see **Figure 6**). These results were confirmed in subgroup analyses of 2, of which 1828 subjects included only women with implantation failure after IVF/ICSI (RR 1.62,95% CI 1.18-2.24, I^2^ = 76%, *P* = 0.0009), and 2157 subjects in the remaining 9 studies underwent hysteroscopy before the first IVF/ICSI procedure (RR 1.61,95% CI 1.27-2.04, I^2^ = 79%, *P* < 0.0001). The quality of evidence was judged to be low (see **Figure 5**).

#### 3.4.3 Abortion rate

**Figure 7:**
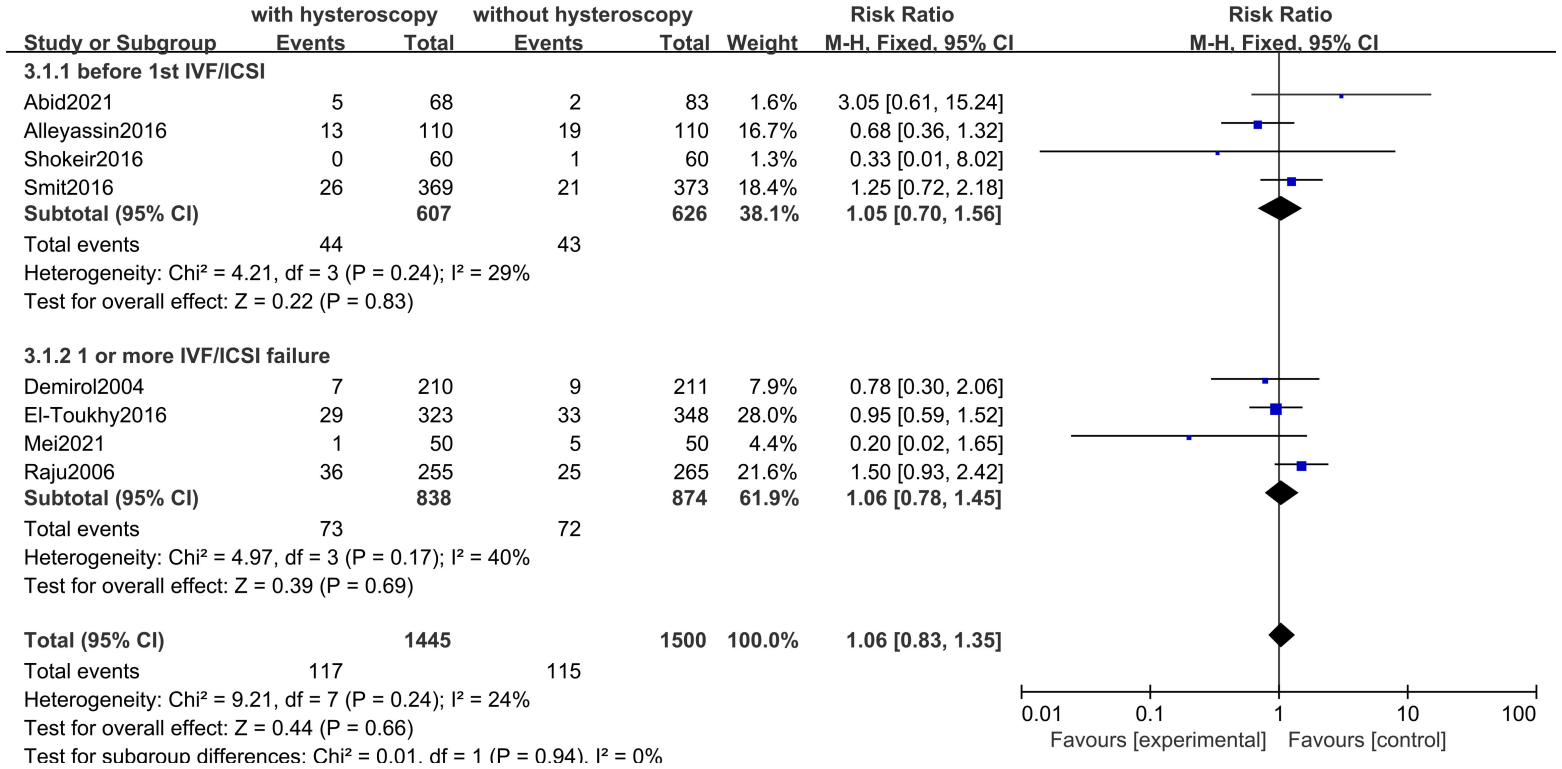
Forest plot of abortion rates.

Eight studies evaluated the impact of hysteroscopy on miscarriage rates, with no significant difference between intervention and control groups (RR 1.06,95% CI 0.83-1.35, I^2^ = 24%, *P* = 0.24), and no between-group difference between the two subgroup analyses of women who underwent hysteroscopy before their first IVF/ICSI procedure and those who had failed implantation after IVF/ICSI (*P* = 0.94)(see **Figure 7**). The quality of evidence was judged to be low (see **Figure 5**).

#### 3.4.4 Complications

A total of 4 studies observed complications, but only one study (25) found one complication in the hysteroscopy team. One patient in the hysteroscope group developed endometritis.

### 3.5 Sensitivity analysis

By excluding individual studies one by one, the changes in the combined effect size of each outcome indicator were observed. The results showed that the combined RR values were similar during the exclusion process, indicating that the results of this Meta-analysis were stable.

## 4 Discussion

### 4.1 Main findings of the study

This study was conducted to evaluate the efficacy of hysteroscopy in the treatment of infertile women. In this study, 14 RCT clinical studies were screened by literature search. After Meta-analysis, it was found that the implementation of hysteroscopy before IVF or ICSI can improve the live birth rate in infertile women in general and can significantly improve the clinical pregnancy rate, regardless of whether these infertile women have taken IVF/ICSI before. Despite the lack of statistical significance, in other words, this may just be a trend, we still saw a slight advantage in pregnancy rates and live birth rates after hysteroscopy prior to the first IVF or ICSI treatment. Meanwhile, our study found that the implementation of hysteroscopy did not have a significant effect on the occurrence of miscarriage rate, and in terms of the observation of complications, there were no facts and evidence of inducing significant complications, with only one case of complication in the hysteroscopy group in one study.

### 4.2 Significance of the study

Embryo implantation is a complex process, and the success of embryo implantation depends on two main conditions: the degree of embryo development and endometrial tolerance. As the application of ART technology expands, endometrial tolerance is widely recognised as a key factor in the success of ART technology. In the past, when technology was not as advanced, we could only rely on ultrasound for indirect knowledge of the uterine cavity, and at that time there were more patients with unexplained infertility. As technology has evolved we have been able to gain a deeper understanding of the internal environment of the uterine cavity, for example, hidden microscopic lesions in the uterine cavity can affect the intrauterine environment and lead to poor pregnancy outcomes (21). For humans, the uterus is their first home. Dr. Linda Bradley at the Cleveland Clinic has said the hysteroscope should be considered the stethoscope for the uterus (8). As is known to us all, hysteroscopy has evolved from a traditional technique for the diagnostic purpose of examining the uterine cavity to a valuable means of simultaneously diagnosing and treating a variety of intrauterine lesions, particularly in a field increasingly focused on female reproduction. Although a variety of tests are now available for non-invasive or minimally invasive examination of reproductive organs such as the endometrium and the uterine cavity, the relative sensitivity and specificity of ultrasound, saline infusion ultrasound and hysteroscopy for the detection of endometriotic lesions in prospective comparisons were 89% and 56%, 91.8% and 60%, and 97.3% and 92%, respectively.

Hysteroscopy has evolved from the traditional art of examining the uterine cavity for diagnostic purposes to a highly valuable modality for diagnosing and (viewing and) treating a wide range of intrauterine pathologies simultaneously. In addition, the local endometrial injury caused by hysteroscopy can improve endometrial blood circulation, increase endometrial tolerance, and improve clinical pregnancy outcomes in IVF-ET patients (27). For infertile women, a successful clinical pregnancy with a healthy delivery is eagerly awaited, and whether or not hysteroscopy can provide such a benefit with improved uterine environment and endometrial tolerance has been concluded differently in different studies. There is also uncertainty as to what point in time hysteroscopy should be performed. Therefore, a systematic evaluation encompassing as many studies as possible is necessary.

According to Stamenov, although hysteroscopy is still an invasive procedure and requires an experienced operator to ensure optimal treatment outcomes, it has the unique advantage of simultaneous visualisation for diagnosis and treatment prior to IVF/ICSI - in short, hysteroscopy should be recommended as a first-line procedure for all female infertility patients (28).

Hysteroscopy has extensive clinical value for a wide range of uterine conditions that are more easily diagnosed and treated symptomatically under visualisation. For example, chronic endometritis is recognised as a potential cause of primary and secondary infertility, and all women with a diagnosis of infertility should be screened for chronic endometritis and treated aggressively. It has been reported that chronic endometritis may be present in more than 60% of women with repeated implantation failures and recurrent miscarriages (29). And endometrial samples collected hysteroscopically show higher specificity and predictive value than other sampling methods (30–32). Similarly, other common disorders such as uterine adhesions, adenomyosis, T-shaped uterus, and mediastinal uterus can be better clinically benefited by hysteroscopy to improve pregnancy outcomes.

This Meta-analysis found that although the use of hysteroscopy prior to IVF/ICSI was not statistically significant in the subgroup analyses for the improvement of live birth rates, this may be due to the fact that fewer live birth rates were observed in the included studies, with a total of only 5 studies observing live birth rates. Overall Meta-analysis of the 5 studies showed that hysteroscopy is effective in improving live birth rates, which gives more confidence and evidence-based clinical evidence. Due to the low cost of observing clinical pregnancy rates, more studies have included indicators of clinical pregnancy rates, and although the definition of clinical pregnancy rates may vary between them, the use of hysteroscopy preoperatively in both first-time preoperative and recurrently failed patients has been shown to provide a clear benefit in improving clinical pregnancy outcomes. We also looked at miscarriage rates and found that hysteroscopy had no significant effect on miscarriage rates in patients with first-time or recurrent implantation failure. Among the 14 included studies, 4 reported the observation results of complications, and only one study found one case of endometritis in the hysteroscopy group. In fact, the incidence of infection caused by hysteroscopy is relatively low, which is reported in the literature to be approximately 0.01% - 0.2%. Therefore, in our study, hysteroscopy is regarded as a better and safer option. Regarding the timing of hysteroscopy, relatively more rigorous studies would mention that, for instance, most studies suggest that it is preferably conducted during the early to mid-follicular phase after menstruation is over. The reason is that during this period, menstruation is clean, the surgical field of vision is better, the endometrium is thinner, facilitating observation. Meanwhile, shallow-positioned polyps disappear spontaneously with the shedding of the endometrium, saving operational steps. Additionally, the risk of pregnancy during the early follicular phase is lower, which is also a necessary consideration for safety. Of course, sometimes, in order to reduce the number of patient visits and relieve patients’ anxious and impatient emotions, it is understandable to relax the requirements of the timing.

Based on the systematic evaluation and literature review, we recommend that hysteroscopy should be actively chosen for infertile patients to clarify the micro and macro environment of the uterine cavity, and to provide timely and effective microscopic management of intracavitary lesions that may affect the rate of conception and live births, with a stable safety profile and definite efficacy in assisting conception.

The limitations of this systematic evaluation are: (1) multiple outcome indicators existed with large heterogeneity among studies, mainly due to inconsistencies in baseline and differences in experimental methods among studies; (2) the sample sizes of the five literatures were small; (3) methodological limitations existed in some of the studies, and there was selective bias and implementation bias; (4) the outcome indicators varied among different literatures; (5) the majority of the literature did not adequately report adverse reactions; (6) analysis of publication bias using a funnel plot revealed poor positional symmetry in the literature, suggesting the possibility of publication bias.

None of the included studies had conducted multicentre large-sample clinical trials, and the methodological quality was generally poor. More high-quality clinical trials should be conducted, random sequence generation and allocation concealment schemes should be developed, blinding should be strictly implemented in accordance with the trial design, quality control during the trial process should be improved, and awareness of clinical trial registration should be raised, and study protocols should be registered in advance in order to standardise the process of study implementation and to provide more reliable study conclusions.

## 5 Conclusion

In conclusion, considering the convenience, practicality, effectiveness and safety of hysteroscopy, choosing hysteroscopy for infertile women can improve clinical pregnancy and live birth rates. because of the poor quality of the included studies, more high-quality RCTs are needed in the future to corroborate the results of this systematic evaluation and to provide high-quality, evidence-based medical evidence for the treatment of infertile women to improve pregnancy outcomes.
